# Reliability analysis of the Ahringer *Caenorhabditis elegans *RNAi feeding library: a guide for genome-wide screens

**DOI:** 10.1186/1471-2164-12-170

**Published:** 2011-03-31

**Authors:** Wubin Qu, Changhong Ren, Yuan Li, Jinping Shi, Jiye Zhang, Xiaolei Wang, Xingyi Hang, Yiming Lu, Dongsheng Zhao, Chenggang Zhang

**Affiliations:** 1 Beijing Institute of Radiation Medicine, State Key Laboratory of Proteomics, Beijing 100850, China; 2 Beijing Institute of Health Administration and Medical Information, Beijing 100850, China

## Abstract

**Background:**

The Ahringer *C. elegans *RNAi feeding library prepared by cloning genomic DNA fragments has been widely used in genome-wide analysis of gene function. However, the library has not been thoroughly validated by direct sequencing, and there are potential errors, including: 1) mis-annotation (the clone with the retired gene name should be remapped to the actual target gene); 2) nonspecific PCR amplification; 3) cross-RNAi; 4) mis-operation such as sample loading error, *etc*.

**Results:**

Here we performed a reliability analysis on the Ahringer *C. elegans *RNAi feeding library, which contains 16,256 bacterial strains, using a bioinformatics approach. Results demonstrated that most (98.3%) of the bacterial strains in the library are reliable. However, we also found that 2,851 (17.54%) bacterial strains need to be re-annotated even they are reliable. Most of these bacterial strains are the clones having the retired gene names. Besides, 28 strains are grouped into unreliable category and 226 strains are marginal because of probably expressing unrelated double-stranded RNAs (dsRNAs). The accuracy of the prediction was further confirmed by direct sequencing analysis of 496 bacterial strains. Finally, a freely accessible database named CelRNAi (http://biocompute.bmi.ac.cn/CelRNAi/) was developed as a valuable complement resource for the feeding RNAi library by providing the predicted information on all bacterial strains. Moreover, submission of the direct sequencing result or any other annotations for the bacterial strains to the database are allowed and will be integrated into the CelRNAi database to improve the accuracy of the library. In addition, we provide five candidate primer sets for each of the unreliable and marginal bacterial strains for users to construct an alternative vector for their own RNAi studies.

**Conclusions:**

Because of the potential unreliability of the Ahringer *C. elegans *RNAi feeding library, we strongly suggest the user examine the reliability information of the bacterial strains in the CelRNAi database before performing RNAi experiments, as well as the post-RNAi experiment analysis.

## Background

Kamath and Ahringer constructed an important RNA interference (RNAi) feeding library of bacterial strains corresponding to roughly 86% of the estimated 19,000 predicted genes in *C. elegans *in 2003 [1-3]. This RNAi feeding library has contributed largely to genome-wide functional studies of the *C. elegans *genes, including embryonic development [4], aging [5,6], fat regulation [7], genome stability [8] and mitochondrial proteins [9], *etc*. However, the library has not been thoroughly validated by direct sequencing [1], and there are potential errors, including: 1) mis-annotation, for example, the clones with the retired gene names should be re-annotated. Retired gene here means that the gene is not a gene today but a transposon or two genes might have been fused together. Although the gene name is retired, the clone is still useful if the strain could hit any gene. 2) non-specific PCR amplification when evaluating the specificity of the primers using MFEprimer [11]; 3) cross-RNAi [2] discovered using the BLAST program [12] to search for the predicted PCR amplicons against the latest version of the *C. elegans *genomic DNA sequence; 4) mis-operation, such as sample loading error [1], *etc*. These errors would potentially attenuate the accuracy of any RNAi experiment. Therefore, it is necessary to evaluate the quality of all bacterial strains in the Ahringer *C. elegans *RNAi feeding library. The virtual qualities of all the bacterial strains in the RNAi library are predicted using a bioinformatics approach. The prediction accuracy was further confirmed by direct sequencing analysis on the 496 bacterial strains. In addition, to make the evaluation results freely accessible from the public domain, a user-friendly database named CelRNAi (http://biocompute.bmi.ac.cn/CelRNAi/) was developed. Moreover, submissions to the database of direct sequencing results or any other annotations on the bacterial strains are allowed and will be integrated into the CelRNAi database to improve the prediction accuracy. Importantly, we also provided five candidate primer sets for each of the unreliable and marginal bacterial strains for users to construct vectors for their own RNAi studies.

## Methods

The 16,256 GenePairs primer pairs for most *C. elegans *genes were downloaded from the web site of Kim Lab at Stanford University (http://cmgm.stanford.edu/~kimlab/primers.12-22-99.html). The *C. elegans *genomic DNA sequence database (version WS202) was downloaded from ftp://ftp.wormbase.org/pub/wormbase/genomes/elegans/sequences/dna/. The unspliced gene database, which contains unspliced gene sequences and annotation information (such as the gene position in *C. elegans *genomic DNA sequences), was downloaded from WormMart (http://caprica.caltech.edu:9002/biomart/martview/). The stand-alone version of MFEprimer (version 1.5, http://biocompute.bmi.ac.cn/MFEprimer/download/) was used to examine the specificity of the primer sets.

An efficient primer set (primer pair) should result in: 1) only a single and unique amplicon for separation by the 0.5-2% agarose gel electrophoresis, and 2) melting temperatures (Tm) of the forward and reverse primers that are close to each other [1]. Therefore, evaluation of the GenePairs primer set basically followed these two criteria: 1) Tm of each primer in the primer set should be greater than 50°C, and the difference between the forward and reverse primers should be less than 3°C. 2) If the primer set results in two or more amplicons, the migration distance (calculated on the basis of mobility of the predicted amplicons) between the expected amplicon and other nonspecific ones on the agarose gel electrophoresis (assuming the gel concentration to be 1%, both here and below) should be greater than M_min_. The M_min _was defined as the minimum distance of two DNA bands in agarose gel electrophoresis, which can be easily determined by the naked human eye. We set M_min _= 2 mm for our evaluation. The distance between amplicon_*i *_and amplicon_*j *_could be calculated according to Formula 1; while the mobility of each amplicon during agarose gels electrophoresis was calculated using Formula 2. This second formula is an experience formula, based on a large number of electrophoresis experiments in our lab (data not shown) [13-15].(1)(2)

Where a = 4.61, b = -0.72, c = 474.65, *Mobility*_*i *_and *Mobility*_*j *_are the mobility of *amplicon*_*i *_and *amplicon*_*j*_, respectively. *Mobility*_*0 *_is the mobility of known *amplicon*_*0*_, such as the 2000 bp DNA fragment of the DNA marker DL2000 (TaKaRa, Japan).

**Figure 1:**
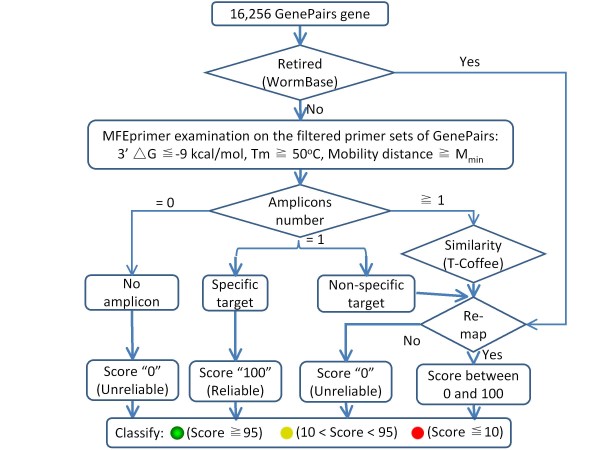
Flowchart of the bioinformatics evaluation process on the Ahringer *C. elegans *RNAi feeding library

Based on the abovementioned descriptions, we performed the reliability analysis on the feeding RNAi library as follows (Figure 1):

1. Search the WormBase gene database (downloaded from WormMart) to check whether the GenePairs name was still alive. If the GenePairs name had been retired, it means that the clone should be re-annotated by remapping to other genes. If the strain could hit other genes, this clone is still useful.

2. Each of the 16,256 GenePairs primer sets was examined by MFEprimer with the following parameters: -e 10000, -W 4, -ppc_cutoff 0.3. The *C. elegans *genomic DNA sequence database was used as target DNA templates for checking the primer's specificity. The initial score for each primer set was set to 100. In the following steps, there were penalties placed on the score until it was zero.

3. Check the Gibbs free energy (ΔG) of the last five residues at the 3' end of each primer. If the value of ΔG was less than -9 kcal/mol, this primer set was discarded because it would probably result in a large number of nonspecific amplicons in this situation. In this case, a score of 0 was also directly assigned to this bacterial strain.

4. Filter the MFEprimer results by Tm range (>50°C): The amplicons with Tm below 50°C were discarded because the annealing temperature for the PCR reaction is 58°C when constructing the RNAi feeding library [1].

5. Filter the MFEprimer results by M_min_: If the primer set resulted in two or more amplicons and the distance of their mobilities on the 1% agarose gel electrophoresis was less than M_min_, we used the program T-COFFEE [16] to score the sequence similarity of these amplicons. The amplicons with similarity scores greater than 95 were considered to be potentially homologous genes, and this primer set was considered to be able to inactivate multiple genes. The penalty was set to [100 - T-COFFEE_score].

6. Find out the genes located in the genomic DNA region where the MFEprimer predicted amplicons were also located. If there was no gene in the region, we used the word "Intergenic" to indicate this case. Sometimes, there were two or more genes in one region, indicating that the amplicon might cover multiple target genes during the RNAi assays.

7. To identify the potential target genes that might be targeted for RNAi by the amplicons which passed the abovementioned criteria, a BLAST search was performed against the *C. elegans *unspliced gene database. Only hits with identities > 80% over a region of at least 200 nucleotides were reported [1-3]. If the potential target genes do not include the gene of the clone, it means that the clone should be re-annotated based on the potential target genes.

8. Mark the bacterial strains in the 384-well plates with road traffic signs and markings: green colored background for reliable strains (score ≥ 95), yellow for marginal strains (10 < score < 95) and red for unreliable strains (score ≤ 10). For the clones (such as retired strains) which do not hit a gene based on the predicted amplicon, the letter "R" with a yellow background will be marked. Other detailed information, such as the MFEprimer report and the BLAST searching results, are also shown in the CelRNAi web site.

## Results

### Reliability analysis of the Ahringer *C. elegans *RNAi feeding library

We use a bioinformatics approach to evaluate all of the bacterial strains (16,256) in the Ahringer *C. elegans *RNAi feeding library (Figure 1). Initially, to determine whether the gene is retired or not, all of the 16,256 gene targets for RNAi of the corresponding bacterial strains were queried in WormBase (http://www.wormbase.org/). Second, all of the PCR primer sets used for preparing the RNAi vectors by amplification of the genomic DNA of target genes in *C. elegans *was evaluated using MFEprimer, followed by careful analysis of the predicted amplicons. Finally, a score (an integer from 0 to 100) was calculated for each of the bacterial strains based on the analysis results (See "Methods" section for details). We grouped the bacterial strains into three categories based on the scores and used road traffic signs and other icons to easily indicate the reliability of each record: 1) Reliable bacterial strains (marked with a green colored background in the web site): score ≥ 95, indicating that the bacterial strains could express the designed dsRNAs; 2) Marginal strains (yellow colored background): 95 > score > 10, indicating that the bacterial strains in this group may express unrelated dsRNAs; 3) Unreliable strains (red colored background): score ≤ 10, suggesting that these bacterial strains probably expressed unrelated dsRNAs or were only empty vectors. For all the clones, if the original gene name (e.g. retired gene name) is not existed in the predicted target gene list, the letter "R" will be marked on the road traffic sign. Results demonstrated that most (98.3%) of the bacterial strains in the library are reliable. However, we also found that 2,851 (17.54%) bacterial strains need to be re-annotated even they are reliable. Most of these bacterial strains are the clones having the retired gene names. The detailed number of bacterial strains of each group is listed in Table 1. All of these results (including detailed analysis information) can be found in Additional file 1.

**Table 1:** Number of the bacterial strains of each group

Group	Number	Sequenced number	Remapped number
Reliable strains	15,979 (98.30%)	286 (1.79%)	2,702 (16.91%)
Marginal strains	249 (1.53%)	186 (74.70%)	122 (49.00%)
Unreliable strains	28 (0.17%)	24 (85.71%)	27 (96.43%)
Total	16,256	496 (3.05%)	2,851 (17.54%)

### Experimental validation of the reliability evaluation on the Ahringer *C. elegans *RNAi feeding library

To confirm our evaluation, the most of marginal and unreliable clones, as well as about three hundred reliable clones were sequenced. The numbers of validated clones of each group were shown in Table 1. The success rate of our evaluation was listed in Table 2. The sequencing evidence showed that in the predicted reliable group, 65.38% (187/286) of the clones are reliable and 26.57% (19.58% retired clones + 6.99% wrong insert clones) of the clones have been remapped to other genes but still useful. However, there are also 8.04% clones are unreliable and should be discarded in the RNAi studies. Because we sequenced almost all the clones in the marginal and unreliable group, it's nonsense to calculate the success rate of our prediction on these two groups. Although we have successfully predicted 91.96% clones in the reliable group, the higher failure rate of 8.04% strongly suggested that it's necessary to sequence all the clones and construct a comprehensive database with sequencing validation evidence support for sharing the information to the scientific community.

**Table 2:** Percentage of the sequenced clones of each predicted group

Predicted records		Records by direct-sequencing-based validation			
	
			Remapped number		
Category	Number	Reliable number			Unreliable number
			Retired	Wrong inserts	
**Green**	286	187 (65.38%)	56 (19.58%)	20 (6.99%)	23 (8.04%)
**Yellow**	186	78 (41.94%)	80 (43.01%)	4 (2.15%)	24 (12.90%)
**Red**	24	4 (16.67%)	16 (66.67%)	1 (4.17%)	3 (12.50%)
**Summary**	496	269 (54.23%)	152 (30.65%)	25 (5.04%)	50 (10.08%)

To analyze the detailed error types of the unreliable types, 30 validated bacterial strains were selected (6 reliable, 18 marginal and 6 unreliable). The GenePairs names for the sequencing results were determined based on a sequence similarity search against the *C. elegans *genomic DNA database (WS202) using the NCBI BlastN program and identifying those with e-values < 10^-6 ^[12]. We carefully analyzed the strains shown in Table 3 and found three types of error: (1) **Remapped**: Strains include Y110A2A_4093.a [No. 26], Y119D3_462.c [No. 28], Y55B1B_119.g [No. 29] and Y67D8A_349.a [No. 30], as shown in Table 3. Because the expected gene has been retired, the target genes of these strains may be localized in intergenic regions or they could be any other genes, as the sequencing results indicated. Other strains described detailed in the following are also remapped to other genes. (2) **Nonspecific primers**: Strains include M142.1 [No. 9], T08D2.2 [No. 10], C03H5.5 [No. 16], C14B1.10 [No. 19], R09A1.2 [No. 21], C49A1.1 [No. 25] and C38H2.2 [No. 27], as shown in Table 3. All of the bacterial strains in this group expressed unrelated dsRNAs and would result in failure of the designed and expected RNAi assay. Three of the bacterial strains (C14B1.10 [No. 19], R09A1.2 [No. 21] and C38H2.2 [No. 27]) expressed intergenic dsRNAs according to the sequence similarity analysis, while the rest expressed other unrelated genes. (3) **Low level errors such as sample loading errors or empty vector constructions**: These strains include F36H12.3 [No. 7], T17A3.3 [No. 11], F32B5.4 [No. 14] and B0554.1 [No. 22], as shown in Table 3. Locations of these strains in the 384-well plate (from the Ahringer *C. elegans *RNAi feeding library) are very close to that of the GenePairs gene which they actually targeted on the basis of direct sequencing results. For example, the strain No. 7 was designed to target the gene at IV-2B04, but the sequencing results indicated that the target is actually the gene located at IV-2D04, showing that the position of this strain shifted vertically only two rows in the same column of the same 384-well plate. Strains of No. 14 and No. 22 have similarly problem, strongly suggesting these strains are probably caused by sample loading errors. Unexpectedly, the GenePairs T17A3.3 [No. 11] contained a vector sequence which failed during plasmid construction, although MFEprimer predicted specific primers. Therefore, this strain may be caused by an unknown operation error.

**Table 3:** Reliability examination of the randomly selected 30 feeding Ahringer *C. elegans *RNAi bacterial strains with different scores

		Information provided by the manufacturer			Information based on direct sequencing analysis				
**Seq. No**.	Reliability score	GenePairs name	GeneService location	Predictable by MFEprimer	GenePairs name	GeneService location	Predictable by MFEprimer	Reliability examination manually	Comment
1	100	F18H3.3	X-6I10	√	F18H3.3	X-6I10	√	√	
2	100	F22E12.4	V-7A24	√	F22E12.4	V-7A24	√	√	
3	100	T07D1.4	X-1J16	√	T07D1.4	X-1J16	√	√	
4	100	K09G1.4	V-6P21	√	K09G1.4	V-6P21	√	√	
5	100	T26G10.1	III-5C07	√	T26G10.1	III-5C07	√	√	
6	100	W01C9.5	II-6A04	√	W01C9.5	II-6A04	√	√	
7	49	F36H12.3	IV-2B04	√	F36H12.17	IV-2D04	**×**	√	Remapped. Probably sample loading error: position of the strain was shifted vertically for two rows in same column of the same plate.
8	48	C17C3.13	II-4D02	√	C17C3.13	II-4D02	√	√	
9	48	M142.1	III-5F06	√	F54F12.2	III-6D13	√	√	Remapped. Nonspecific amplicon and may be prevented by MFEprimer evaluation on the primer set at first.
10	46	T08D2.2	V-13E02	√	W07B8.1	NA (not in the library)	√	√	Remapped. Nonspecific amplicon and may be prevented by MFEprimer evaluation on the primer set at first.
11	44	T17A3.3	III-1E13	√	NA	NA	**×**	**×**	Empty vector construction based on direct sequencing
12	42	F58B4.1	V-7B19	√	F58B4.1	V-7B19	√	√	
13	36	F48G7.5	V-1G12	√	F48G7.5	V-1G12	√	√	
14	32	F32B5.4	I-1I14	√	C45E1.1	I-1M14	×	√	Remapped. Probably sample loading error: position of the strain was shifted vertically for four rows in same column of the same plate.
15	30	K11H12.1	IV-1A12	√	K11H12.1	IV-1A12	√	√	
16	28	C03H5.5	II-1O03	√	Y48G8AL.11	NA (not in the library)	√	√	Remapped. Nonspecific amplicon and may be prevented by MFEprimer evaluation on the primer set at first.
17	27	C32E8.3	I-1H21	√	C32E8.3	I-1H21	√	√	
18	26	F10G8.2	I-5C13	√	F10G8.2	I-5C13	√	√	
19	24	C14B1.10	III-1J10	√	NA	NA	√	**×**	Nonspecific amplicon (Intergenic region [438973..440037] in chromosome I) and may be prevented by MFEprimer evaluation on the primer set at first.
20	23	F59H6.8	II-2E10	√	F59H6.8	II-2E10	√	√	
21	22	R09A1.2	V-1F13	√	NA	NA	√	**×**	Nonspecific amplicon (Intergenic region [3064499..3065944] in chromosome V) and may be prevented by MFEprimer evaluation on the primer set at first.
22	22	B0554.1	V-1K09	√	Y39D8A.1	V-1K07	**×**	√	Remapped. Probably sample loading error by the manufacture: position of the strain was shifted horizontally for two columns in same row of the same plate.
23	20	C45G7.5	IV-1N13	√	C45G7.5	IV-1N13	√	√	
24	14	W02G9.1	V-2M18	√	W02G9.1	V-2M18	√	√	
25	4	C49A1.1	I-7I17	√	Y48G8AL.13	NA (not in the library)	√	√	Remapped. Nonspecific amplicon and may be prevented by MFEprimer evaluation on the primer set at first.
26	0	Y110A2A_4093.a	II-9H01	**×**	K02F6.9	NA (not in the library)	√	√	Retired gene name and remapped to the new gene.
27	0	C38H2.2	III-5F21	**×**	NA	NA	**×**	**×**	Nonspecific amplicon (Located in chromosome X according to the BLAST similarity search) and may be prevented by MFEprimer evaluation on the primer set at first.
28	0	Y119D3_462.c	III-7A23	**×**	Y119D3A.2	NA (not in the library)	√	√	Retired gene name and remapped to the new gene.
29	0	Y55B1B_119.g	III-7G04	**×**	Multiple targets predicted	NA	**×**	√	Retired gene name and remapped to multiple new genes.
30	0	Y67D8A_349.a	IV-8B22	**×**	NA	NA	**×**	**×**	Retired

Note: NA represents for "not available".

### Development of the CelRNAi database as a complementary tool to the Ahringer *C. elegans *RNAi feeding library

To make our evaluation results accessible for the public domain, a user-friendly database named CelRNAi (http://biocompute.bmi.ac.cn/CelRNAi/) was developed as a valuable complement for the Ahringer *C. elegans *RNAi feeding library. This database provides the prediction information for all bacterial strains as well as the direct-sequencing information for the experimentally identified bacterial strains. To create the database, we first stored all the evaluation information in a SQLite (http://www.sqlite.org/) database. Second, a user-friendly front-end web interface was developed with Python/CGI (http://www.python.org/). The CelRNAi database contains 52 virtual 384-well plates (distributed on six chromosomes), corresponding to the real plates in the Ahringer *C. elegans *RNAi feeding library. Each well in the virtual plates has been clearly marked by one of the road traffic signs and markings (green, red and yellow colored background and letters), with the exception of the blank wells. These signs and markings legibly indicate the predication result for the bacterial strains in the wells. For example, the red colored background indicates the bacterial strain in this well is unreliable, while green and yellow represent reliable and marginal, respectively. Additionally, there is a hyperlink on each sign, and markings linking to detailed evaluation information for each of the bacterial strains. The linked information is self-explanatory and contains most of the essential information necessary to show the quality of the strains, such as the MFEprimer report and NCBI Blast hits. We also provided a downloadable Excel file (Additional file 1) which contains brief accounts of all of the evaluation results available from the abovementioned web sites.

The CelRNAi website supports four types of searchable items, including GenePairs name, Main name, WormBase gene ID and the GeneService location on the 384-well plate. In addition, submissions to the database of direct sequencing results or any other annotations for the bacterial strains are welcome and will be integrated into the CelRNAi database to improve the prediction accuracy. Accordingly, the bacterial strains which have been annotated by sequencing results will be marked by three modified signs and markings. First is a red colored background with a "wrong cross" to indicate the strain is unreliable based on supporting experimental evidence. Second is a green colored background with a "right tick" to indicate the strain is reliable, also based on experimental evidence. Third is a letter "R" with green background to indicate the clones have been remapped to other genes based on the sequencing result. Moreover, we provide five candidate primer sets for each of the unreliable and marginal bacterial strains (with the exception of the retired ones) for the users to construct alternative vectors for their own RNAi experiments. Importantly, another web server (http://biocompute.bmi.ac.cn/MPprimer/worms_primer_design.html) is also provided for the users to design specific primers for the *C. elegans *genes of interest, especially useful for genes having no corresponding RNAi strains in the library.

## Discussion

When evaluating the Ahringer *C. elegans *RNAi feeding library, we found that many of the primer sets can amplify homologous genes (such as F31A3.4 and F31A3.2). In some cases, silencing of these homologous genes has its own advantages, as these genes are usually partially redundant in function or have specialized functions [2,3]; this is especially true when the desired phenotype is hard to observe unless all of the homologous genes are inactivated. However, based on the rigorous bioinformatics analysis, there are also some primer sets which can amplify completely different genes at the same time, although there is low or even no significant sequence similarity among these genes. We therefore used T-COFFEE [16], a multiple sequence alignment tool, to perform sequence alignment of these amplicons for scoring the sequence similarity. If the score reported by T-COFFEE is higher than 95, these amplicons are considered to be potentially homologous genes.

Another issue which should be mentioned is the fact that there are 2,851 (17.54%) strains have been remapped to other genes, and most of them have the retired gene names. However, these clones are still useful because they could hit other genes. The users should check the potential target genes at first before utilizing these strains for RNAi assay. This assertion is mainly based on updates of the genomic DNA sequence and genetic information annotation (http://www.wormbase.org/).

Although ePCR [17] can be used to predict the amplicon(s) of the PCR primers against the DNA template (*C. elegans *genomic DNA sequence) and was used during the preparation of the Ahringer *C. elegans *RNAi feeding library [1-3], it is insufficient to evaluate the specificity of PCR primers. The reasons have been discussed in our previous work on MFEprimer [11]; for example, there are several factors influencing the primer's specificity, such as melting temperature (Tm), stability at the 3' end of the primers and sequence similarity (binding sites between the primer and the DNA template). Therefore, we strongly suggest that users run the MFEprimer program to examine the PCR primers to improve the specificity of the PCR reactions in similar studies. In addition, to make certain the specificity of the PCR primers, stricter parameters should be used while running the MFEprimer program, for example, W (word size) = 4, e (expect value) = 10,000 [11].

## Conclusions

We performed a reliability analysis on the Ahringer *C. elegans *RNAi feeding library, which contains 16,256 bacterial strains, using a bioinformatics approach. Although the results demonstrated that most (98.3%) of the bacterial strains in the library are reliable, we found that 2,851 (17.54%) bacterial strains need to be re-annotated even they are reliable. Most of them are the clones having retired gene names. Besides, 28 strains are grouped into unreliable category and 226 strains are marginal because of probably expressing unrelated dsRNAs. The accuracy of the prediction was confirmed by direct sequencing of 496 selected bacterial strains. To share with the public domain with our evaluation results, a freely accessible database named CelRNAi (http://biocompute.bmi.ac.cn/CelRNAi/) was developed as a valuable complement for the feeding RNAi library by providing the predicted information on all bacterial strains as well as the direct-sequencing information for the identified bacterial strains. We strongly suggest the user examine the reliability information of the bacterial strains in the CelRNAi database before performing RNAi experiments, as well as the post-RNAi experiment analysis.

## Authors' contributions

WQ wrote the main manuscript, performed the evaluation and constructed the web site. CR, YLi, JS and JZ carried out the directly sequencing experiments. XW and DZ helped to construct the web server. XH and YLu participated in programming and debugging. CZ designed and supervised the project, finalized the manuscript. All authors read and approved the manuscript.

## Supplementary Material

Additional file 1 **Summary of the evaluation results of all the bacterial strains (16,256) in the Ahringer *C. elegans *RNAi feeding library**. An Excel file which contains brief accounts of all the bacterial strains (16,256) in the Ahringer *C. elegans *RNAi feeding library. Information of the three groups (reliable, marginal and unreliable) are also shown in this file. Click here for file
